# Application of the Intervention Mapping protocol to develop Keys, a family child care home intervention to prevent early childhood obesity

**DOI:** 10.1186/s12889-015-2573-9

**Published:** 2015-12-10

**Authors:** Courtney M. Mann, Dianne S. Ward, Amber Vaughn, Sara E. Benjamin Neelon, Lenita J. Long Vidal, Sakinah Omar, Rebecca J. Namenek Brouwer, Truls Østbye

**Affiliations:** Department of Community and Family Medicine, Duke University Medical Center, 2200 W Main St, Box 104006, Durham, NC 27705 USA; Center for Health Promotion and Disease Prevention, University of North Carolina at Chapel Hill, 1700 Martin L. King Jr. Blvd, CB 7426, Chapel Hill, NC 27599-7426 USA; Department of Nutrition, Gillings School of Global Public Health, University of North Carolina at Chapel Hill, 2207 McGavran-Greenberg Hall, CB 7461, Chapel Hill, NC 27599-7461 USA; Department of Health, Behvaior and Society, Johns Hopkins Bloomberg School of Public Health, Johns Hopkins University, 624 N Broadway, HH755, Baltimore, MD 21205 USA; Duke Office of Clinical Research, Duke University Medical Center, 2424 Erwin Rd, DUMC, Box 2713, Durham, NC 27705 USA

**Keywords:** Childhood obesity, Family child care, Intervention mapping, Diet, Physical activity

## Abstract

**Background:**

Many families rely on child care outside the home, making these settings important influences on child development. Nearly 1.5 million children in the U.S. spend time in family child care homes (FCCHs), where providers care for children in their own residences. There is some evidence that children in FCCHs are heavier than those cared for in centers. However, few interventions have targeted FCCHs for obesity prevention. This paper will describe the application of the Intervention Mapping (IM) framework to the development of a childhood obesity prevention intervention for FCCHs

**Methods:**

Following the IM protocol, six steps were completed in the planning and development of an intervention targeting FCCHs: needs assessment, formulation of change objectives matrices, selection of theory-based methods and strategies, creation of intervention components and materials, adoption and implementation planning, and evaluation planning

**Results:**

Application of the IM process resulted in the creation of the Keys to Healthy Family Child Care Homes program (Keys), which includes three modules: Healthy You, Healthy Home, and Healthy Business. Delivery of each module includes a workshop, educational binder and tool-kit resources, and four coaching contacts. Social Cognitive Theory and Self-Determination Theory helped guide development of change objective matrices, selection of behavior change strategies, and identification of outcome measures. The Keys program is currently being evaluated through a cluster-randomized controlled trial

**Conclusions:**

The IM process, while time-consuming, enabled rigorous and systematic development of intervention components that are directly tied to behavior change theory and may increase the potential for behavior change within the FCCHs.

## Background

In the United States (US), one in four preschool-age children is overweight or obese [1, 2] and children often carry this excess weight into adolescence and adulthood [3, 4]. Being overweight in childhood is associated with both immediate and long-term adverse health outcomes [5–16]. Because almost half of those who become obese in childhood are already overweight by the age of five [1], obesity prevention requires early intervention to promote healthy diet and physical activity habits before excess fat has been deposited.

Child care is an important setting for targeting early obesity prevention efforts [17, 18] given that approximately 60 % of US children under the age of six are in some type of non-parental care, spending on average 25 h or more there each week [19–21]. Child care providers can play a central role in shaping healthy diet and physical activity behaviors in children. Children may consume 50 to 70 % of their daily food intake while in child care [22]; however, meals and snacks currently served in child care do not meet USDA guidelines for key foods like vegetables, whole grains, and milk [23]. Personal characteristics of the director, such as BMI, are associated with quality of foods served (e.g., canned fruit, whole grains, and low-fat milk) [24]. Furthermore, nearly half the variation in children’s physical activity between 9 am and 5 pm can be explained by the child care facility they attend [25, 26]. This variability in physical activity has been attributed to the variety of portable play equipment, outside time, staff behavior, and training [27]. This evidence highlights the importance of the child care setting and provider in shaping children’s weight-related behaviors and, hence, the importance of targeting them in interventions. Providers have the ability to create supportive environments that serve healthy meals and snacks, provide opportunities and encouragement for active play, and serve as adult role models of healthy behaviors.

The family child care home (FCCH) is a child care setting deserving of more attention. FCCHs serve approximately 1.5 million children in the US [21]. FCCHs have elements of formal child care, in that they are small businesses that provide care for young children and must conform to state and local licensing and administrative regulations. However, FCCHs are smaller than centers and operate in the provider’s own home. Regulations for FCCHs are different, and in most cases less stringent, than those for centers and can even differ within the same state [28–30] [31, 32]. States tend to have more regulations governing foods and beverages served to children in care in centers [29]. Similarly, regulations are more robust for physical activity in centers, compared to family child care homes [29, 31, 32]. While there have been several intervention studies targeting centers, very few intervened in FCCHs.

In response to this need, we developed a comprehensive intervention to engage FCCH providers as “champions of child health.” The Intervention Mapping (IM) [33] process was selected to guide the development of a new FCCH-based early childhood obesity prevention intervention. The IM approach includes a systematic, six-step, iterative method to intervention development, implementation, and evaluation [33]. Each step is designed to guide developers through producing an intervention and evaluation rooted in theory and evidence. The process is iterative and allows for adaptation between steps. The steps include: (1) needs assessment, (2) formulation of change objectives matrices, (3) selection of theory-based methods and strategies, (4) creation of intervention components and materials, (5) development of an adoption and implementation plan, and (6) development of the evaluation plan. We chose to apply this process because it provided an organized and structured approach to intervention development. The process helped ensure that the final intervention integrated the lessons learned from formative work, remained grounded in theory, created content that would drive change in targeted behaviors, and incorporated appropriate measures into the evaluation. The purpose of this paper is to describe the application of the IM process and the resulting Keys to Healthy Family Child Care Homes (Keys) intervention.

## Methods

Support for the development, implementation and evaluation of the Keys program is provided by the National Institutes of Health (R01HL108390) as part of a five-year intervention study. The first year of the project was dedicated to development of intervention and evaluation protocols and materials using the six steps of IM. The resulting protocols were approved by the Institutional Review Boards of the University of North Carolina at Chapel Hill and Duke University Medical Center.

### Step 1: Needs assessment

Development of the Keys program began with a needs assessment, which was informed by an informal review of the literature and two pilot studies. The literature review was used to identify existing health-related research efforts being conducted in FCCHs, as well as the behavioral and environmental factors in child care that contribute to obesity. Articles were identified using the researchers’ existing knowledge of the literature (including recent systematic reviews of nutrition and/or physical activity interventions in young children), a search of scientific articles in PubMed, and a search of gray literature in Google. Various combinations of search terms were employed, including child obesity, family child care, child care, participation/enrollment, and interventions.

Two pilot studies provided additional data about FCCH providers to help supplement the limited research available on this population. The first pilot study (conducted in April-May 2010) consisted of an online survey of a convenience sample of 87 FCCH providers about foods served for meals and snacks, time provided for physical activity (i.e., outside time, teacher-led activities, and media use), communication with parents around diet and physical activity, personal health behaviors, and business practices (e.g., numbers enrolled, fees, staffing). The second pilot study (conducted in March-May 2011) collected detailed information about child and FCCH provider behaviors, and examined the acceptability and feasibility of an eight-week FCCH-based intervention with five FCCH providers. The participating FCCH providers were a convenience sample recruited from a 20 mile radius of the project office. These FCCHs likely served families with at least a moderate income since only one of the five FCCHs accepted child care subsidies (a marker for serving low-income families). From these five FCCHs, 15 parents agreed to sign consent and allow measures to be collected on their children (60 % of enrolled children). Measures, taken at baseline only, assessed child diet (Dietary Observation in Child Care, DOCC [34]) and physical activity behaviors (accelerometry), the FCCH nutrition and physical activity environments (Environment and Policy Assessment and Observation, EPAO [35], modified for use in FCCHs), and provider personal diet (dietary screener) and physical activity (accelerometry) behaviors. The pilot intervention included a self-assessment of current practices, a home visit, an in-person workshop, and three follow-up coaching phone calls. These components drew from previous intervention materials developed by the investigative team for child care providers and families [36–38], but were adapted for use with FCCH providers. At the conclusion of the program, providers rated the usefulness and feasibility of the intervention, and their confidence in adopting the recommended changes.

### Step 2: Formulation of change objectives matrices

**Fig. 1:**
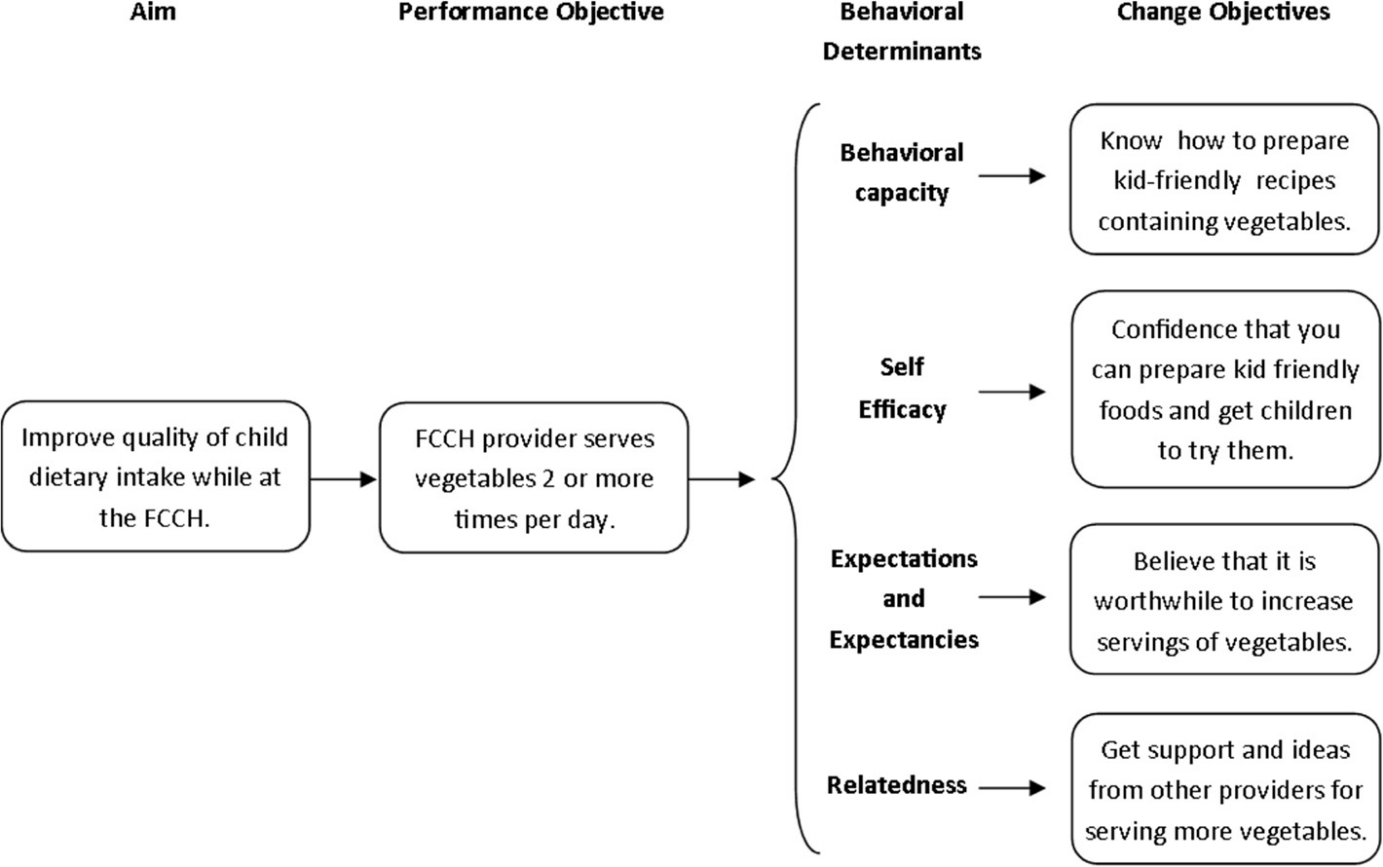
Defining determinants of behavioral change in the IM process in the Keys intervention

Change objective matrices were then developed for performance objectives, theory-based behavioral determinants, and change objectives. The change objective matrices were created around the study’s two primary aims: to improve children’s diet quality and to increase their moderate-to-vigorous physical activity (MVPA) while in the FCCH. Performance objectives reflect changes in FCCH provider behaviors that would support improvement of children’s diet and physical activity behaviors. Selection of these performance objectives was informed by the literature review, and investigators’ own prior child care-based intervention research [36]. Next, determinants of behavioral change were identified. Social Cognitive Theory [39] and Self-Determination Theory [40] informed the selection of the putative psychological constructs to support change to FCCH provider behavior to meet the performance objectives. Social Cognitive Theory and Self-Determination Theory were selected, as both have proven to be useful in the development of effective nutrition and physical activity behavior change interventions in children [41, 42]. Social Cognitive Theory identifies several constructs that influence learning and the adoption of new health behaviors. Self-Determination Theory focuses explicitly on what motivates people to change behaviors. Specifically, the constructs identified were competence and behavioral capacity (knowledge and skills), self-efficacy, expectations and expectancies (attitudes and beliefs), autonomy, and relatedness (social support). Finally, change objectives were created that specified how the theory-based behavioral determinants were translated to support each performance objective. Figure 1 illustrates the process used to identify individual change objectives.

### Step 3: Selection of theory-based methods and strategies

The next step was to select theory-based methods and strategies to achieve these changes. In this process, Social Cognitive Theory [39] was carried forward from the previous step to guide selection of behavior change strategies. Self-Determination Theory [40] provided an understanding of determinants around motivation for change; however, Motivational Interviewing techniques [43, 44] offered more specific strategies and hence was used to guide selection of strategies. Self-Determination Theory and Motivational Interviewing share the same fundamental assumptions, and it is recommended that the two are most useful when used in tandem [45]. Lastly, we incorporated adult learning principles since the intervention would be delivered to adults who care for children. Evidence suggests that actively involving adults in acquiring, using, evaluating, and reflecting on new skills and knowledge is the most effective way for adults to learn [46].

### Step 4: Creation of intervention components and materials

Once these theoretical underpinnings of behavior change determinants and behavior change strategies were identified, the next step was the creation of tangible intervention components. Lessons learned from the second pilot study were used to guide development of intervention components and materials. While many of the components from the pilot were retained, the pilot had significantly less content and lacked scripts and guides for coaches that were needed to ensure consistent delivery of messages and behavior change techniques. In addition, a community advisory group was convened to gather feedback on acceptability of the intervention’s main messages (i.e., performance objectives from step 2) as well as draft materials and intervention components (step 4). Members of the community advisory group included local FCCH providers (including two providers who participated in the second pilot study), as well as professionals who provide technical assistance to FCCHs, and parents with children enrolled in an FCCH. We convened available members three times for group discussion and also collected feedback through email.

### Step 5: Development of an adoption and implementation plan

Development of the adoption and implementation plan was largely completed in tandem with step four because implementation planning is highly dependent on what has to be disseminated and to whom. Results from pilot studies and feedback from the community advisory group were instrumental during this phase of development, particularly in reference to participant burden.

### Step 6: Development of the evaluation plan

Lessons learned and decisions made throughout the intervention development process guided the development of the evaluation plan. The performance objectives, behavioral determinants, change objectives, and behavior change theories were all explicitly documented, which in turn informed the selection of appropriate measures that could assessment outcomes as well as hypothesized pathways of change. Additionally, the clearly defined implementation plan (developed from step 5) was used to inform the development of the process evaluation, including the assessment of reach, dose delivered, dose received, and intervention fidelity.

## Results and discussion

### Step 1: Findings from the needs assessment

The literature review conducted as part of the needs assessment highlighted the dearth of published research conducted in FCCHs. However, there were several studies examining the FCCH environment that suggested FCCHs may be an even more critical setting to target for obesity prevention efforts. A study by Benjamin and colleagues [47] found that time spent in informal child care settings (but not centers) during infancy was positively associated with BMI z-scores at one and three years of age. Little is known about the foods eaten by children in FCCHs; however, a study by Trost and colleagues [48] of FCCH providers noted several nutrition practices needing improvement. Specifically, only 13 % served only low-fat or skim milk to children two years and older, only 44 % limited 100 % fruit juice to less than once a day, and 56 % allowed unhealthy foods for celebrations. Temple and colleagues [49] found that children in FCCHs engaged in 14 min of MVPA in an 8-h day, which is much lower than estimates for children in centers (38-56 min of MVPA) [25, 50]. Furthermore, two separate studies have found that children in FCCHs watch significantly more television compared to those in center-based care [51, 52]. These findings may be explained in part by FCCH providers’ lack of training. Trost and colleagues [48] found that less than 50 % of FCCHs providers received annual training on nutrition or physical activity. These studies identify numerous areas for improvement of healthy behaviors in FCCHs.

The pilot survey of FCCH providers provided a glimpse into the practices of FCCH providers and reiterated the importance of targeting FCCH providers in childhood obesity prevention efforts. Results from self-administered questionnaires suggest that providers did not have an accurate perception of their own health as demonstrated by the fact that 75 % rated their health as “very good” or “excellent” yet 74 % were overweight or obese. On average, providers served 5.4 (±2.7) children in their home, of which 23 % were 1 year old, 19 % were 2 years old, and 24 % were 3-4 years old. About 90 % of providers reported that they felt “quite a bit” or “a lot” of responsibility for getting children to eat healthy foods and be physically active, yet their self-reported practices demonstrated that they provide meals that were high in fat and children engaged in too much sedentary time. More than half of providers served fried foods at least once a week, roughly three quarters did not regularly serve lean meats, and almost half served primarily whole milk to children two years and older. In addition, more than a third had the television on for more than 30 min during the day.

The second pilot study allowed a more detailed examination of child and FCCH provider behaviors and an examination of the acceptability and feasibility of an eight-week intervention. Child participants ranged from 1 to 5 years of age (27 % were 1 year, 33 % were 2 years, and 40 % were 3–5 years). Healthy Eating Index scores, calculated from DOCC data, were lower than recommended, with scores averaging 64.3 (range 52.8–71.5) out of 100. Accelerometer data showed that children, on average, accumulated only 20.2 min of MVPA but 215 min of sedentary time while at the FCCH. Measures of the FCCH physical activity and nutrition environment collected during in-home observations also showed great need for improvement. Scores from the EPAO were 11.3 (out of 20) for the nutrition environment, and 9.12 (out of a possible 20) for the physical activity environment. Similar to the previous pilot survey, providers had poor awareness of their own health. In their self-administered questionnaires, providers generally rated their own health as “very good” or “excellent,” but they reported poor health behaviors. Specifically, four of the five providers were overweight or obese, ate on average only 3.1 cups of fruits and vegetables per day, and obtained on average only 13.4 min of MVPA during 7.8 h of monitoring. These data confirmed the need for improvement in several areas.

The second pilot study also showed that providers responded well to the intervention program. The intervention integrated three topics: provider practices that foster healthy habits in children, personal health behaviors of the provider, and smart business practices. Integrating these three topics allowed us to address the FCCH’s social and physical environment related to children’s physical activity and nutrition, as well as a number of factors that impact the FCCH’s ability to implement and sustain these changes. Providers found all three topics to be helpful, and all were confident or very confident that they had learned new skills to help improve in each of these areas. While feedback from participants was positive, the pilot study was limited in its duration (eight weeks). The coach delivering the intervention in this pilot had only a fixed number of contacts through which to deliver the intervention, including one in-home visit, one short workshop, and three brief coaching calls. All topics were addressed during each contact, which limited the depth of content delivered.

### Step 2: Development of change objective matrices

**Fig. 2:**
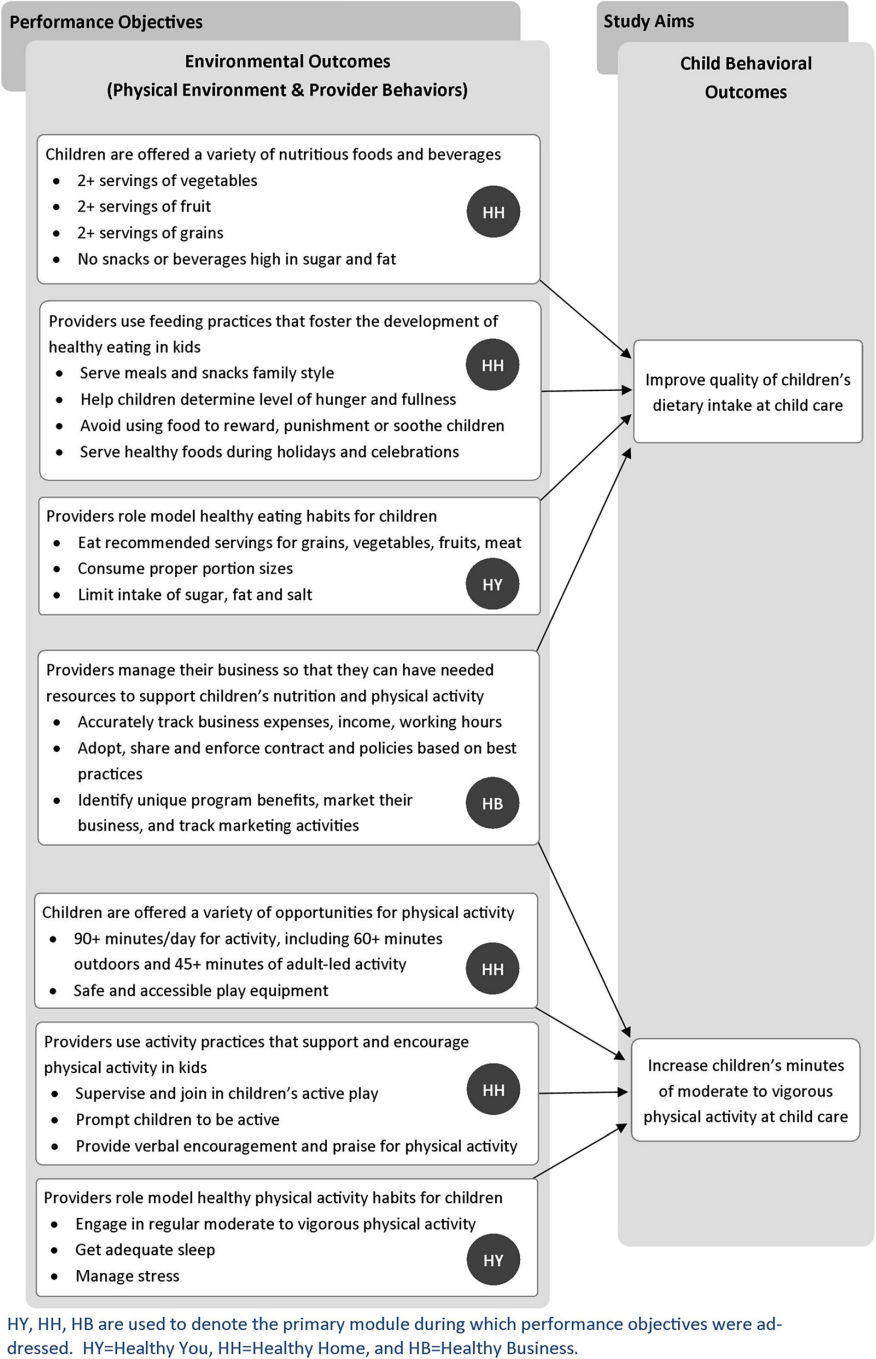
Key’s performance objectives and thier relation to study aims

Findings from the literature review and the pilot studies informed the development of the change objective matrices. Through an iterative process, this information was used by researchers to develop three matrices. In the first matrix, performance objectives targeted the providers’ own health behaviors so they may become healthy role models for the children in their care. In the second matrix, performance objectives focused on creating an FCCH environment that would foster healthy physical activity and eating habits in children. In the third matrix, performance objectives targeted the provider’s business practices in order to help reduce stress and overcome business-related barriers to change. In each matrix, program aims and corresponding performance objectives were used as row headers. Behavioral determinants, specifically knowledge, skills, self-efficacy, attitudes, and social support, and autonomy became the column headers in each matrix. Specific change objectives were then identified for each cell within the matrix. Figure 2 presents the project’s performance objectives, which focus largely on environmental outcomes (defined as the FCCH physical environment and provider behaviors), and how they align with the study aims and desired behavioral changes in children. Table 1 then illustrates this process using one sample performance objective from each module: the provider health matrix (Healthy You), the home environment matrix (Healthy Home), and the FCCH business matrix (Healthy Business).

**Table 1:** Examples of behavioral change objective matrices from each used module used in the Keys intervention

Program aim	Performance objective	Behavioral determinants					
		Knowledge	Skills	Self-efficacy	Attitudes	Social support	Autonomy
Healthy You							
Providers consume high quality diets	PO 1	K.1.1	S.1.1	SE.1.1	A .1.1	SS.1.1	Au.1.1
	Providers limit their intake of sugar, fat and salt	Know the effects of high amounts of sugar and fat in the diet	Ability to read food packaging labels to evaluate quality of food products	Feel confidence in ability to limit sugar, fat, and salt	Believe that it is worthwhile to limit sugar, fat, and salt	Awareness that others struggle with limiting sugar, fat, and salt	Choose to decrease the amount of sugar, fat, and salt in personal diet
		K.1.2	S.1.2	SE.1.2		SS.1.2	
		Know the effects of high amounts of sodium in the diet	Ability to identify and prepare healthy alternatives for meals, snacks, and beverages	Feel confidence in ability to overcome barriers to eating a healthy diet and limit sugar, fat, and salt		Locate and interact with other providers who are trying to limit sugar, fat, and salt	
		K.1.3					
		Awareness of common foods and beverages that contain high amounts of sugar, fat, and salt					
Healthy Home							
Increase amount of physical activity children accumulate while in child care	PO 2	K.2.1	S.2.1	SE. 2. 1	A.2.1	SS.2.1	Au.2.1
	FCCH daily schedules include recommended time for various physical activity for children	Know that the recommended amount of physical activity for children is 120 min per day	Ability to lead physical activity for children two times per day	Feel confident in ability to help children enjoy physical activity	Believe that physical activity is important for children	Get support and ideas from other providers on incorporating physical activity into regular activities	Choose to provide time for various activity throughout the day
		K.2.2	S.2.2	SE.2.2			
		Know that physical activity should not be withheld for bad behavior	Ability to plan outdoor active play time two times per day	Feel confident in the ability to not limit physical activity for			
			S.2.3				
			Ability to				
			reward good behavior with physical activity	bad behavior			
Healthy Business							
Providers have and enforce a comprehensive contract and set of policies based on best business practices	PO 3	K.3.1	S.3.1	A.3.1	SS.3.1	PO 3	K.3.1
	Have a current and comprehensive contract based on best business practices	State the important components of a contract	Feel confident in ability to create a contract for providing services as an FCCH	Express positive attitudes toward having a contracts and set of policies	Ask other providers about their policies (but not their program costs due to legality)	Have a current and comprehensive contract based on best business practices	State the important components of a contract
				A.3.2			
				Express belief that you have the power to set or change policies			

FCCH, family child care home; MVPA, moderate-to-vigorous physical activity

### Step 3: Selection of theory-based methods and strategies

Once matrices were developed, theory-based methods and strategies were identified that would help providers achieve the change objectives. Social Cognitive Theory [39] and Self-Determination Theory [40] had been identified by the team as useful theories to guide intervention development and hence were used to inform selection of these strategies. This process was aided by lists of behavior change strategies included in the IM protocol [53]. These lists specify methods to use for specific types of behavior change, associated theory, and parameters of use.

The methods of behavior change identified as most relevant included: persuasive communication, guided practice, self-evaluation, autonomy building, physiological and affective change tools, and active learning. Once these methods were chosen, the research team developed strategies (individual activities) that met the definition of the method but also employed adult learning principles to actively engage providers in acquiring, using, evaluating, and reflecting on new skills and knowledge. The Keys intervention study aims and performance objectives are presented in Fig. 2. Table 1 shows how the multiple methods and strategies employed by the Keys intervention work toward meeting each behavioral change objective and shows the theory that supports the use of each method. The examples correspond to the performance objectives and behavioral change objectives in Table 2.

**Table 2:** Examples of methods and strategies matrices from one module used in the Keys intervention

Performance objective	Intervention component	Method	Strategy	Targeted behavioral change objectives	Theoretical basis
PO2	Workshop 2:	Persuasive communication	Power point presentations	K.2.1, K.2.2	SCT
*Increase the amount of physical activity children accumulate while in child care*	Healthy Home	Guided practice	Stretching activity (Learning active transitions)	S.2.1, SE.2.2	SCT
			Adult-led activity examples: Activity cards, yoga card exercises, animal grab bag	S.2.1, SE.2.2	SCT
		Active learning	Best practices brainstorming Group work – Physical activity	K.2.1, K.2.2,	SCT
				SE.2.1, SE.2.2	
				A.2.1	
				SS.2.1	
	Coaching	Environmental evaluation	Healthy home self-assessment	K.2.1	SCT
			Motivational interviewing during workshop and individual coaching sessions	K.2.1, K.2.2,	SCT
				S.2.1, S.2.2, S.2.3	
				SE.2.1, SE.2.2	
				A.2.1	
				SS.2.2	
		Autonomy building	Motivational interviewing during individual coaching sessions	K.2.1, K.2.2,	SDT
				S.2.1, S.2.2, S.2.3	
				SE.2.1, SE.2.2	
				A.2.1	
				SS.2.2	
				Au.2.1	
	Educational Tool Kit	Physiological and affective change tools	Children’s books promoting physical activity	S.2.1, S.2.2, S.2.3	SCT
			Poly-spots (Activity spots)	S.2.1	SCT
		Persuasive communication	Keys branded educational materials	K.2.1, K.2.2,	SCT
				S.2.1, S.2.2, S.2.3q	

FCCH, family child care home

Additionally, Motivational Interviewing was chosen as a behavior change strategy because it is centered on participants and requires both self-evaluation and autonomy building, falling in line with both Social Cognitive Theory (self-evaluation) and Self-Determination Theory (autonomy building). Table 2 demonstrates how the Keys intervention employed multiple methods and strategies to work towards each behavioral change objective and shows the theory that supports the use of each method. The examples correspond to the performance objectives and behavioral change objectives in Table 1.

### Steps 4 and 5: Creation of program components, materials, and implementation plan

The resulting Keys program was designed around three modules: Healthy You, Healthy Home, and Healthy Business; each module corresponded to content on one of the three behavioral change matrices. In the first module, Healthy You, content focuses on provider personal nutrition, physical activity, sleep and stress behaviors. This topic was selected for the first module as it was intended to build provider interest and motivation by focusing on their own health needs first. Behavioral recommendations were based on adult guidelines [54]. The second module, Healthy Home, focuses on how the FCCH can support healthy nutrition and physical activity in children. Best practice recommendations were based on previous work by investigators on the Nutrition and Physical Activity Self-Assessment for Child Care (NAP SACC) program [36] as well as recent updates to this program for Go NAP SACC. The third module, Healthy Business, focuses on record keeping, contracts and policies, and marketing. Content for this module came directly from Tom Copeland’s curricula about the business of family child care [55–57]. The community advisory group confirmed the importance of all three modules and was agreeable to the proposed ordering of content.

While topics addressed in each module vary, they include the same intervention components—an in-person group workshop, a set of educational materials and tool-kit resources, and four one-on-one coaching contacts. The workshop is three hours in length and designed to be primarily educational, building providers’ behavioral capacity (knowledge and skills). Workshops also include opportunities to reflect on their current behaviors, set goals and plan for change, and build social support between providers as they exchange ideas (relatedness). Educational binders and tool kit resources are disseminated during workshops and help reinforce the content delivered during workshops. The binders were professionally illustrated and produced to ensure a high-quality appearance.

Tool-kits provide practical resources (e.g., pedometers, re-useable water cups, child sized divided plates, poly spots, diet and physical activity related books, etc.) to support provider efforts to create healthier environments. The community advisory group confirmed the importance of the in-person group workshop, but predicted some difficulties with attendance given providers’ busy schedules. Additionally, they generally had positive feedback on the materials they were asked to review by email.

Coaching sessions are designed to last 30–45 min and employ Motivational Interviewing techniques. A general guide was developed for these coaching contacts to ensure some consistency, but the nature of Motivational Interviewing is that it is a participant-led process that allows for autonomy building by letting the participant, rather than the coach, lead the conversation and set goals. During each contact, the health behavior coach works with the provider to review current goals, assess progress toward goals, problem-solve around any barriers that might be encountered, and revise action plans as needed. Motivational interviewing strategies, such as using open ended questions and change talk are used to increase participant’s intrinsic motivation. Additionally, reflective listening, affirmations, and autonomy support are used to recognize participant strengths, progress, and personal choice and investment in moving towards their goals. Participants are provided with tracking sheets and encouraged to self-monitor on a daily or weekly basis to help them stay on track.

Full program implementation of all three modules lasts eight to nine months, with approximately ten weeks being dedicated to each module. The workshop is used to launch each module and introduce providers to the content. Coaching contacts follow, spaced two or three weeks apart. The initial coaching contact is done in-person at the FCCH, and the remaining contacts are conducted by phone. All intervention components are delivered by health behavior coaches trained on content and in motivational interviewing techniques. The community advisory group recognized that the length of the program represented a long commitment for providers; however, they felt that offering credits toward continuing education would provide a good incentive and help address this potential barrier.

### Step 6: Development of an evaluation plan

To evaluate the efficacy of Keys, a cluster randomized controlled trial (children are clustered within the FCCH) with a pre-test post-test design. The study will enroll a total of 150 FCCHs. Following completion of baseline data collection, FCCHs are randomly assigned 1:1 into either the intervention or control arm. At the conclusion of the program, all FCCHs complete follow-up data collection.

Those in the intervention arm receive the Keys intervention. For comparison, an attention control group is used. The attention control receives a similarly structured program, but content for all three modules focuses on only business-related topics. These topics align with those covered in the Healthy Business module of Keys, but each topic is covered in greater depth. For the control arm, we wanted to include a topic that would be of interest to the majority of family child care home providers but also not target or elicit a nutrition or physical activity improvement. Because family child care homes operate on tight budgets and are at-risk for closure due to finances, we included the Healthy Business module in both the control and intervention arms.

The study has two primary outcomes: children’s dietary intake quality and minutes of MVPA while at the FCCH. Children’s diet quality is calculated with a Healthy Eating Index score [58, 59] which is based on dietary intake data collected with the DOCC [34]. Children’s MVPA is calculated based on three days of accelerometer wear using an ActiGraph GT3X+ monitor and applying age-appropriate cut-points [60]. In addition, secondary outcomes include child BMI, provider health behaviors (weight, diet) [61], and physical activity (accelerometer), and FCCH nutrition and physical activity environment, policies, and practices (via the EPAO) [35]. Details of these methods are described elsewhere [62].

Evaluation efforts also include process evaluation measures, specifically the assessment of reach, dose delivered, dose received, and intervention fidelity. To assess reach, a detailed recruitment tracking database was created. For each community that is targeted for recruitment, the database records the total number of licensed FCCHs present, as well as the number screened, not interested, not eligible (along with reasons for ineligibility), consented, and measured. These data will allow assessment of what percent of eligible FCCHs were reached and to explore differences between participants and non-participants. To evaluate implementation (dose delivered, dose received, and intervention fidelity), a separate intervention tracking database was created. The database captures attempts to deliver intervention components (dates of workshops, coaching attempts), completion of each component by participant and date of completion. These data will allow evaluating of delivery of the intended intervention (dose delivered), participant completion rates (dose received), and fidelity to protocol (completion within the specified time frame). In addition, coaching contacts are recorded and reviewed to assess the use of motivational interviewing techniques and coverage of intervention topics. Lastly, evaluation surveys were created for each module (for workshops and coaching contacts) to capture an overall rating (poor to excellent), ratings for coaches’ ability to maintain engagement, be responsive to questions, offer support, and build motivation, and perceptions of burden.

This paper presents the process used to develop Keys, an early childhood obesity prevention intervention and its evaluation. The Keys intervention program is unique in that it is designed for use in FCCHs, a child care environment rarely targeted for intervention. Development employed the full six-step IM process, including needs assessment, formulation of change objectives matrices, selection of theory-based methods and strategies, creation of intervention components and materials, development of an adoption and implementation plan, and development of the evaluation plan. The result is a comprehensive intervention program that builds on the best available evidence from current literature and integrates theoretical models. The IM process took 1 year to complete, not including the two pilots that were conducted before the larger study was funded. While time-consuming, the systematic approach to intervention and evaluation development ties the program more closely to behavior change theory and well-defined behavior change strategies, which in turn can increase chances of actual positive behavior change [41, 63].

One major theme that emerged throughout the IM process was that FCCH providers are a group with particularly high needs when it comes to promotion of healthy child diet, physical activity, and weight – current practices appear to be very poor and needs for technical assistance and support are great. The literature suggested that children enrolled in FCCHs may have increased risk for obesity, which may be due in part to the providers’ poor nutrition and physical activity practices and the lack of training in nutrition and physical activity. Our pilot survey also demonstrated that many providers feel a great deal of responsibility for getting children to eat healthy foods and be physically active, yet they are still providing foods that are high in fat and too much sedentary time. Furthermore, the pilot of the eight-week intervention suggested that even a half-day workshop was able to increase provider confidence and self-efficacy (based on self-report). The community advisory group recognized that there was a need for FCCH providers to come together, as they do not generally do so already. However, motivating providers to come together requires that they understand why it is important and how it is going to affect their business.

Another key theme that emerged was the importance of addressing FCCH providers’ needs around business and finances. Child care programs, particularly FCCHs, are businesses with low profit margins and provider incomes are very low. Data from the pilot survey showed that they charged just over $600 per month per child (on average), and cared for about five children at any given time. Furthermore, 68 % of providers had an annual income of less than $50,000 per year (meaning that they make below the median income for this area). During the pilot intervention, workshop evaluations highlighted providers’ interest in the business topics. Two of the five providers noted the business content as the most helpful information presented; taxes and record keeping were specific topics that they wanted future iterations of the program to address. The community advisory group also reiterated the importance of the business component noting that financial cost and administration time of any recommended changes must be recognized and minimized. It is critical for the success of the Keys program that this feedback be incorporated in a meaningful way. Hence, the final intervention has an entire module dedicated to “Healthy Business.” Additionally, financial considerations are integrated into the earlier modules where appropriate (e.g., how to save money purchasing healthy foods and what types of physical activity supports for children are tax deductible). To help ensure that the intervention addressed the specific needs of FCCH businesses, we also employed an expert consultant (Tom Copeland) with extensive FCCH experience.

While IM does provide a rigorous and useful process for intervention development, we encountered some challenges during implementation. One challenge was the lack of previous research in FCCHs, which provided limited evidence-based strategies. Some lessons were applied from interventions conducted in centers (e.g., having some in-person group workshops but being flexible with scheduling, assembling a community advisory group to provide feedback on intervention approaches); however, FCCHs are different settings with their own unique challenges and opportunities. The pilot studies helped inform the larger intervention; however, both had limited sample sizes and were drawn from convenience samples from central North Carolina. Given the challenges encountered during the needs assessment, a community advisory group provided insight and guidance. Given the diversity in membership (FCCH providers, parents, and members of community partnerships that provided technical assistance to FCCH providers), the number of providers at each meeting was generally limited to two or three. Therefore, the size and composition of the group may have limited the amount and breadth of feedback obtained from one of the most important sources—the FCCH providers themselves.

Going forward, researchers planning to use the IM process may benefit from a slight modification in the approach around selecting theory-based methods and strategies (step 3). Currently IM suggests that selection of behavior change strategies should be based on theory and includes a set of clearly identified behavior change techniques associated with each theory. However, Michie and colleagues [64] have integrated behavior change techniques from six classification systems and created a taxonomy that offers clear terminology and operationalized definitions that may be more widely agreed upon and easy to recognize. Much of the Keys intervention development (steps 2-6) occurred in 2012, and this taxonomy was not yet available at that time. However, integrating use of this taxonomy into the IM process would allow more systematic identification of behavior change strategies employed in a study and facilitate comparison of the strategies used across studies.

## Conclusions

The IM process encouraged the development of an intervention closely tied to theories of behavioral change. In Keys, the IM process of development was closely linked to these behavior change theories, and mechanisms of change were easily identified, assessed, and replicated. Additionally, the methods and strategies could be reassessed and changed where needed. Here, IM was a feasible tool for intervention development to create an evidence-based and theory-driven intervention applied to a novel setting. Subsequent steps will include the rigorous evaluation of the impact of the Keys intervention and dissemination of intervention results to help inform future interventions targeting FCCHs.

## Abbreviations

(FCCH): Family child care home
(IM): Intervention mapping
(MVPA): Moderate-to-vigorous physical activity
(EPAO): Environment and Policy Assessment and Observation
(DOCC): Diet Observation in Child Care
(NAP SACC): Nutrition and Physical Activity Self-Assessment for Child Care
